# Supplementary material for: Acute kidney injury and hyponatremia in hospitalized patients with rotavirus infection
Source: PLoS One. 2026 Feb 19;21(2):e0326830. doi: 10.1371/journal.pone.0326830 (PMC12919823; doi:10.1371/journal.pone.0326830)
Supplement: S2 Table — (DOCX) [file pone.0326830.s002.docx]

**Suppl. Table S2: Multivariate logistic regression analysis of risk factors for the development of AKI.**

|  | **Univariate Logistic Regression** | | **Multivariate Logistic Regression** | |
| --- | --- | --- | --- | --- |
|  | OR (95%CI) | p value | OR (95%CI) | p value |
| **Age** | 1.01 (0.99,1.02) | 0.4 |  |  |
| **Age group ≥70 years** | 1.23 (0.75, 2.04) | 0.4 |  |  |
| **Sex, male** | 1.12 (0.71, 1.76) | 0.6 |  |  |
| **CKD** | 2.66 (1.65, 4.33) | **<0.001** | 3.29 (1.92, 5.77) | **<0.001** |
| **Charlson comorbidity index** | 1.07 (0.91, 1.26) | 0.4 |  |  |
| **Community- acquired infection** | 6.97 (3.97, 12.9) | **<0.001** | 8.00 (4.43, 15.3) | **<0.001** |
| **Co-morbidities** |  |  |  |  |
| **Diabetes mellitus n (%)** | 1.31 (0.82, 2.07) | 0.3 |  |  |
| **Hypertension n (%)** | 2.26 (0.94, 5.63) | 0.071 |  |  |
| **Heart failure n (%)** | 0.83 (0.47, 1.45) | 0.5 |  |  |
| **Malignoma n (%)** | 0.56 (0.23, 1.27) | 0.2 |  |  |
| **Coronary heart disease n (%)** | 1.17 (0.70, 1.94) | 0.6 |  |  |
| **Peripheral artery disease n (%)** | 0.83 (0.32, 2.00) | 0.7 |  |  |
| **Renal replacement therapy n (%)** | 0.92 (0.27, 2.81) | 0.9 |  |  |
| **Abbreviations: CI, confidence interval; OR, odds ratio** | | | | |
