# Supplementary material for: Acute kidney injury and hyponatremia in hospitalized patients with rotavirus infection
Source: PLoS One. 2026 Feb 19;21(2):e0326830. doi: 10.1371/journal.pone.0326830 (PMC12919823; doi:10.1371/journal.pone.0326830)
Supplement: S3 Table — (DOCX) [file pone.0326830.s003.docx]

**Suppl. Table S3: Multivariate logistic regression analysis of risk factors for in-hospital mortality.**

|  | **Univariate Cox-Regression** | | **Multivariate Cox-Regression** | |
| --- | --- | --- | --- | --- |
|  | HR (95%CI) | p value | HR (95%CI) | p value |
| **Age** | 1.05 (1.01,1.10) | **0.019** | 1.06 (1.01, 1.11) | 0.010 |
| **Sex, male** | 0.75 (0.34,165) | 0.5 |  |  |
| **CKD** | 1.92 (0.86, 4.30) | 0.11 |  |  |
| **Charlson Comorbidity Index** | 0.91 (0.68, 1.21) | 0.5 |  |  |
| **Community- acquired infection** | 2.87 (1.23, 6.69) | **0.015** | 2.48 (0.92, 6.70) | 0.074 |
| **Co-morbidities** |  |  |  |  |
| **Diabetes mellitus n (%)** | 0.70 (0.3, 1.63) | 0.4 |  |  |
| **Hypertension n (%)** | 0.90 (0.21, 3.85) | 0.9 |  |  |
| **Heart failure n (%)** | 0.63 (0.26, 1.52) | 0.3 |  |  |
| **Malignoma n (%)** | 1.88 (0.74, 4.79) | 0.2 |  |  |
| **Coronary heart disease n (%)** | 0.58 (0.22, 1.54) | 0.3 |  |  |
| **Peripheral artery disease n (%)** | 0.27 (0.04, 1.99) | 0.2 |  |  |
| **Renal replacement therapy n (%)** | 0.90 (0.28, 2.87) | 0.9 |  |  |
| **AKI** | 2.63 (1.20, 5.74) | **0.016** | 2.03 (0.83, 4.97) | 0.12 |
| Abbreviations: CI, confidence interval; HR, hazard ratio | | | | |
