# Supplementary material for: Acute kidney injury and hyponatremia in hospitalized patients with rotavirus infection
Source: PLoS One. 2026 Feb 19;21(2):e0326830. doi: 10.1371/journal.pone.0326830 (PMC12919823; doi:10.1371/journal.pone.0326830)
Supplement: S1 Fig — The day of rotavirus diagnosis is day 0. Means with 95% confidence intervals (black line: no AKI, red line = AKI, green area = normal range). (PPTX) [file pone.0326830.s004.pptx]

## Slide 1
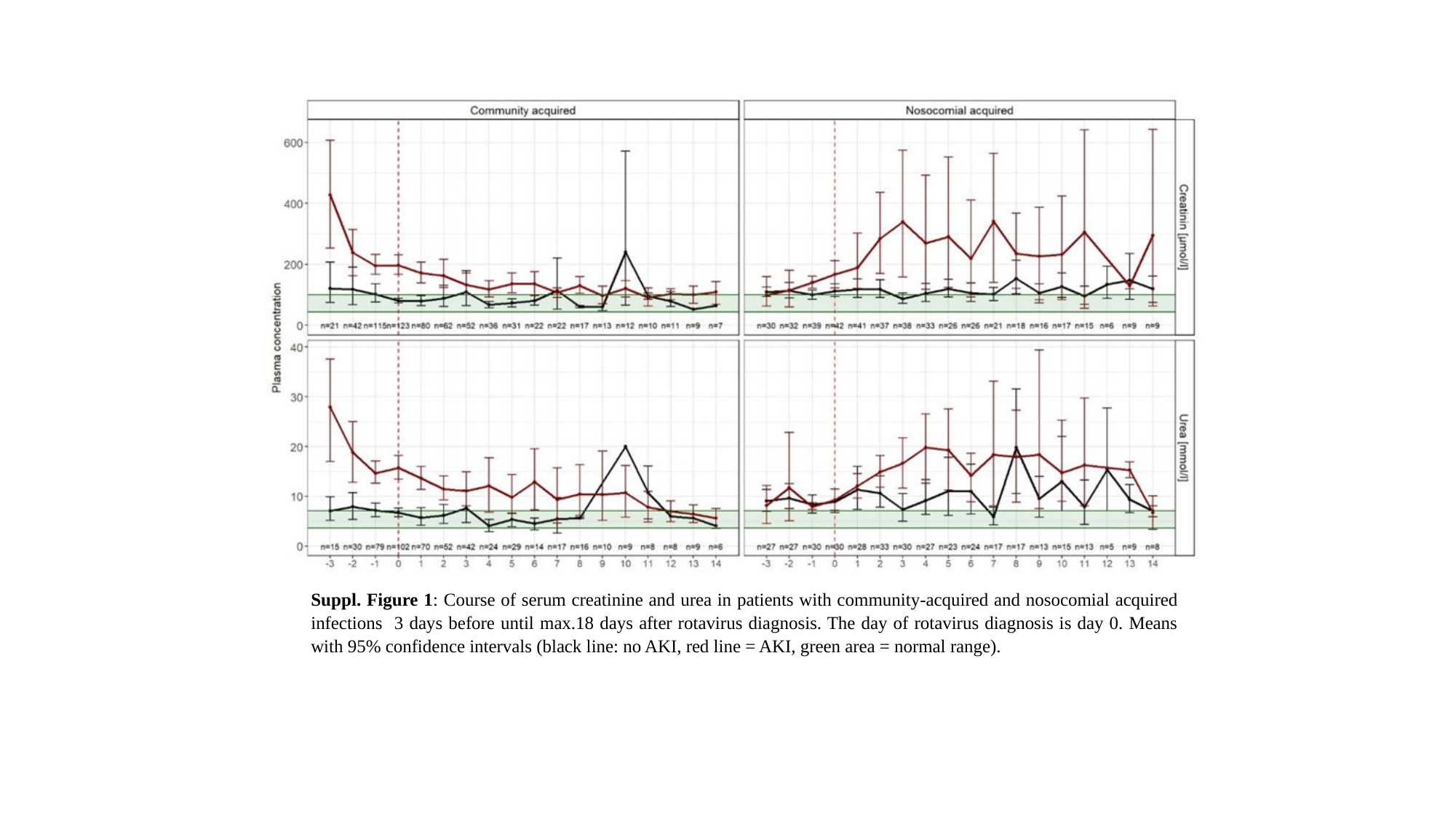

Suppl. Figure 1: Course of serum creatinine and urea in patients with community-acquired and nosocomial acquired infections 3 days before until max.18 days after rotavirus diagnosis. The day of rotavirus diagnosis is day 0. Means with 95% confidence intervals (black line: no AKI, red line = AKI, green area = normal range).
